# 582. Feeling the Vibes: An Investigation Into Resident Physician Antibiotic Prescribing Practices

**DOI:** 10.1093/ofid/ofae631.177

**Published:** 2025-01-29

**Authors:** David Dickson, Jaime Jordan, Tara Vijayan

**Affiliations:** Stanford, Redwood City, CA; UCLA, Los Angeles, California; University of California Los Angeles, David Geffen School of Medicine, Los Angeles, CA

## Abstract

**Background:**

Efforts to improve inpatient antibiotic prescribing are limited by a lack of insight into the heuristics and thought processes that influence the complicated decisions around antimicrobial use. We aimed to explore antimicrobial therapeutic decision making among resident physicians at a large academic health system.

**Figure:**
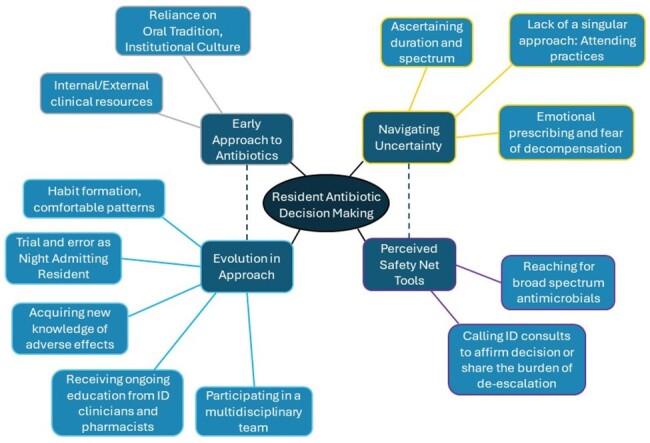

**Figure 1.** Thematic map of resident trainee antibiotic decision making process

**Methods:**

We performed a qualitative study with a constructivist paradigm employing semi-structured, in-person focus groups of Internal Medicine interns and residents at the UCLA Ronald Reagan and Santa Monica Medical Centers from December 2023 through January 2024. Sessions were recorded, auto-transcribed, and edited for accuracy. Two researchers independently performed a thematic analysis of three distinct focus group transcripts.

**Results:**

25 residents participated in a total of three focus groups. Residents consistently identified a clear general approach to prescribing antibiotics, including triaging critical illness and identifying the following: the presence of infection; the source of infection; the antibiotic that covers the likely pathogens; and co-morbid conditions and risk factors. Two broad themes regarding initial acquisition of an approach to antibiotics emerged: institutional culture or “oral tradition,” and internal and external clinical resources. A major challenge in therapeutic decision-making centered around navigating uncertainty (Figure 1). Comments on the inconsistency of clinical reasoning and “vibes-based” prescribing were common. Residents identified certain safety net strategies to mitigate the anxieties associated with this uncertainty (Figure 1). Resident confidence improved over time via habit formation and trial and error, particularly when working independently as the Night Admitting Resident. ID physicians and pharmacists provided a model approach for therapeutic reasoning and supported residents to take risks they were otherwise reluctant to take (such as de-escalation).

**Conclusion:**

Despite articulating a systematic approach to antimicrobial prescribing, resident therapeutic decision-making remains subject to many vulnerabilities, including emotional prescribing. A reliance on the ID consult as a safety net may account for increases in consult volume and requires further investigation.

**Disclosures:**

**All Authors**: No reported disclosures

